# The #StopAsianHate Movement on Twitter: A Qualitative Descriptive Study

**DOI:** 10.3390/ijerph19073757

**Published:** 2022-03-22

**Authors:** Jiepin Cao, Chiyoung Lee, Wenyang Sun, Jennie C. De Gagne

**Affiliations:** 1 School of Nursing, Duke University, Durham, NC 27710, USA; jiepin.cao@duke.edu (J.C.); jennie.degagne@duke.edu (J.C.D.G.); 2 School of Nursing & Health Studies, University of Washington Bothell, Bothell, WA 98011, USA; 3 Department of Education, Culture & Society, The University of Utah, Salt Lake City, UT 84112, USA; wenyang.sun@utah.edu

**Keywords:** Asian Americans, data mining, social media, qualitative research

## Abstract

Evidence-based intervention and policy strategies to address the recent surge of race-motivated hate crimes and other forms of racism against Asian Americans are essential; however, such efforts have been impeded by a lack of empirical knowledge, e.g., about racism, specifically aimed at the Asian American population. Our qualitative descriptive study sought to fill this gap by using a data-mining approach to examine the contents of tweets having the hashtag #StopAsianHate. We collected tweets during a two-week time frame starting on 20 May 2021, when President Joe Biden signed the COVID-19 Hate Crimes Act. Screening of the 31,665 tweets collected revealed that a total of 904 tweets were eligible for thematic analysis. Our analysis revealed five themes: “Asian hate is not new”, “Address the harm of racism”, “Get involved in #StopAsianHate”, “Appreciate the Asian American and Pacific Islander (AAPI) community’s culture, history, and contributions” and “Increase the visibility of the AAPI community.” Lessons learned from our findings can serve as a foundation for evidence-based strategies to address racism against Asian Americans both locally and globally.

## 1. Introduction

Asian Americans (AAs), the fastest-growing racial/ethnic group in the United States [1], have been threatened by a surge of discrimination and hate crimes since the onset of the COVID-19 pandemic. Anti-Asian hate crimes increased by 77% in 2020, according to Federal Bureau of Investigation data [2]. In a recent survey of 352 AAs, more than half reported having experienced racist incidents, and about one third expressed fear that they would be threatened or attacked [3]. Although the World Health Organization has warned that such terms are stigmatizing and should be avoided as they can lead to racial profiling [4], the continued use of the racist terms “Chinese virus” and “China virus” is the reason most often cited by people of Asian descent for the rise of hate crimes against them [3]. The surge and escalation of anti-Asian hate crimes, in particular, the killing of an 84-year-old Asian man in San Francisco and the mass murder of six Asian women in Atlanta in 2021 has led to a nationwide counter movement with an online presence. One example is the launch of the #StopAsianHate tag on Twitter, which serves as a forum for the expression of thoughts and opinions regarding Asian hate (i.e., hatred of Asian people).

The alarming number of racist hate crimes and incidents and their detrimental effects on the health and safety of AAs highlight the urgent need to respond to anti-Asian American violence by addressing the roots of structural racism against AAs. AAs are challenged in their daily lives by various forms of discrimination and othering experiences [4], including perpetual foreigner stereotyping [5] and model minority stereotyping [5,6]. A meta-analysis conducted by Lee and Ahn [7] found that the experience of racial discrimination was strongly associated with depression and anxiety among Asians living in America and other Westernized cultures. Efforts to address racist violence and antagonism against people of Asian descent must reach across systems at the individual, community, institutional, and societal levels, including education, health care, and criminal justice systems. Although efforts have been made to address this critical issue (e.g., the COVID-19 Hate Crimes Act and the U.S. Department of Health and Human Services initiative on Asian American, Native Hawaiians, and Pacific Islanders [WHIAANHPI]), evidence-based policies and interventions are in their infancy due to limited knowledge about the roots and effects of racism against AAs.

An examination of #StopAsianHate tweets presents a viable starting point to enhance understanding of the lived experience of racism against AAs in order to inform evidence-based strategies to address it [8]. Emerging as a new medium for social movement, Twitter has been used as a platform for discussion and activism regarding diverse social issues. Tweeters can connect with others, engage in conversations, share resources with community members, and share similar experiences on a specific topic. Historically, Twitter has allowed the research community to learn about other online social movements, such as the #Metoo movement [9,10] and #BlackLivesMatter [11,12].

### 1.1. Twitter as an Important Source for Research Data

Twitter, since its inception in 2006, has stood out from other social media networks as a unique source for big data because of the real-time nature of its content and the ease of accessing and searching its publicly available information [13]. In 2009, Twitter’s tagline was changed from “What are you doing?” to “What’s happening?”. This seemingly simple shift was a critical move for the social media platform and was a strategic effort to further differentiate itself from others, such as Facebook and Instagram [13]. One recent survey completed in 2021 found that nearly half of U.S. adult tweeters responded that their use of the site had increased their understanding of social events over the past year [14]. Crucially, discussions about race and racism on Twitter are extremely rich—Twitter provides a space for people to publicly express their ideas and viewpoints on social issues related to race, which is not often available in the “off-line” world and other platforms [15]. In addition, the Twitter Developer Platform allows users to capture data in a convenient way where multiple tools are available such as the publicly available Twitter Application Programming Interface (Twitter API) [13]. These contributions provide a rich source of data on this sensitive topic and allow researchers to track, capture, and analyze tweeters’ responses and activities [13]. In particular, digital data mining of tweets has been used to help identify hot topics and blind spots within the field of racial studies, including in AA Studies [16,17]. Considering the aforementioned features, Twitter is the most appropriate platform to understand public disclosure toward the #StopAsianHate movement.

### 1.2. Objective and Hypotheses

Utilizing Twitter as an activist platform, the present study examines the contents of tweets with the hashtag #StopAsianHate in the context of racism against AAs by analyzing social media posts (i.e., tweets) using thematic analysis. Although AAs and Pacific Islanders are distinct groups, we used Asian American and Pacific Islander (AAPI) in results and discussion wherever appropriate as the two communities tend to be referenced together in tweets and other data sources.

## 2. Materials and Methods

### 2.1. Study Design and Data Source

The current study uses a qualitative descriptive design. We extracted data from Twitter using the Twitter API. Twitter API is an immensely useful tool for data mining [18]. The interface allows for the performance of complex queries, such as extracting tweets related to a certain topic made during a specific period of time. It stores the extracted data, which includes all available fields for each tweet from the Twitter data stream, in a structured format, such as a multi-tabbed Microsoft Excel^®^ (http://www.microsoft.com, accessed on 2 June 2021) workbook. It also returns a summary spreadsheet for hashtag entries, mentions, URLs, users, retweets, favorites, and specific words [19]. Twitter API can be implemented in different programming languages, and we chose BirdIQ (https://birdiq.net, accessed on 2 June 2021). This software uses Twitter Search API to extract historical data (please see more details in Bartra et al. [18]). It is a user-friendly interface providing different filters for information extraction as desired, such as tweets that contain a particular hashtag. In this study, we collected tweets containing the hashtag #StopAsianHate that were published from 20 May to 4 June 2021. This start date was made in consideration of President Joe Biden’s historically significant signing of the COVID-19 Hate Crimes Act on 20 May 2021. This legislation to combat hate crimes against AAPIs is an important step toward addressing structural racism, the underlying root of hate. We did not limit the geographic locations of the tweets. A total of 31,665 tweets from the 14-day period were extracted and were exported into Excel^®^.

### 2.2. Data Screening and Data Mining

**Figure 1:**
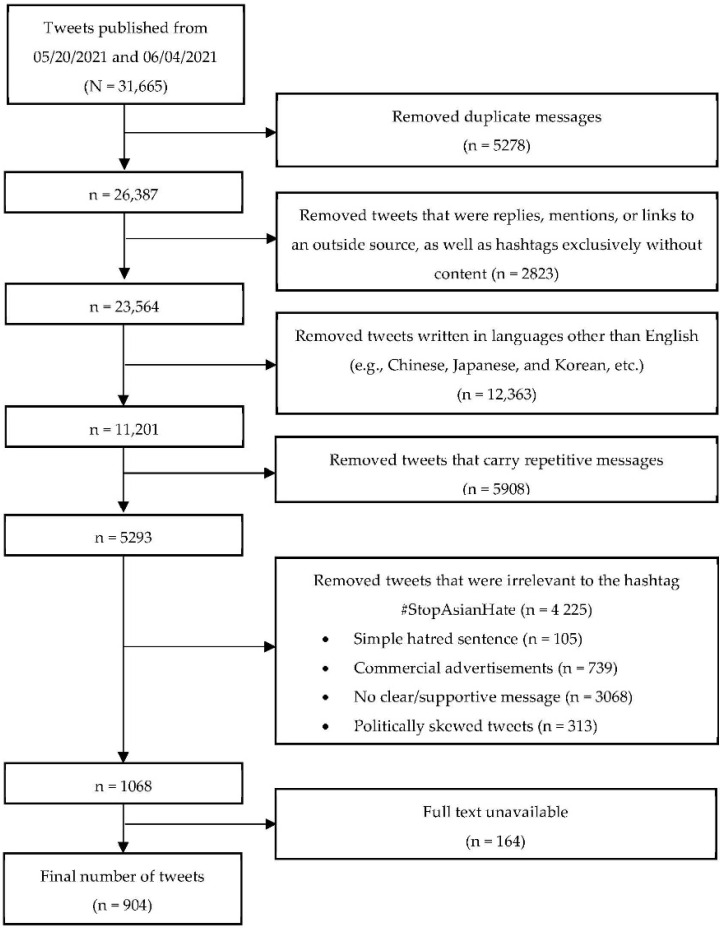
Data mining process using Excel.

From the 31,665 tweets initially extracted, a total of 5278 duplicates were removed before further eligibility screening was conducted. Retweets were not used in our analysis. The first and second authors further examined and excluded tweets that (1) were only replies, mentions, or links to an outside source, as well as exclusively hashtags without any content; (2) were written in a language other than English (e.g., Chinese, Japanese, Korean); (3) carried repetitive messages; or (4) were irrelevant to the hashtag #StopAsianHate (e.g., commercial advertisements). BirdIQ allows users to collect only the first 140 characters of data in each tweet, so if tweets were cut off, the coding team followed available URLs to access the full original text. Using this process, we found that the full texts of 164 tweets were unavailable. Finally, 904 tweets met the eligibility of our study and were exported into NVivo 12.0 (QSR International Pty Ltd., Melbourne, Australia) for data analysis. We only exported the text of the tweets; other digital media information (e.g., images, audios, videos) was not exported. The data screening process is presented in Figure 1.

### 2.3. Data Analysis and Rigor

The data were analyzed using thematic analysis, which provides a solely qualitative, detailed, and nuanced account of data [20]. We did not apply prior codes or coding schemes to the coding process because the empirical evidence on this topic is limited. No theoretical lens was applied as our analysis was based on an inductive approach. Two coders (J.C. and C.L.) analyzed 50% of the tweets independently and developed their own codes. Then the initial codebook was developed after comparing the individual codes and resolving discrepancies by discussion. The coding team used the initial codebook to code the tweets and made revisions to the codebook as appropriate until consensus was reached and no significant changes to the codes were necessary. With the revised codebook, the coding team coded the remaining tweets until information saturation was reached without new codes emerging. The coding team reviewed all codes and combined them into themes after several discussions.

Several strategies were used to ensure the trustworthiness of this study in terms of its confirmability, dependability, credibility, and transferability [21]. For confirmability, the team reported the study methods in detail and supported results with example tweets. To ensure dependability, we used an audit trail to keep track of all decisions made by the team throughout data analysis. We reached an agreement on the results and interpretations through multiple discussions to ensure credibility. The limitations of the samples are discussed to allow for transferability evaluation by the reader.

### 2.4. Ethical Considerations

To protect the privacy of all tweeters and their personal information, Twitter allows its data to be shared in the form of a spreadsheet that only includes Tweet IDs [22]. The study team, however, undertook additional measures to ensure the privacy and digital rights of users. First, we only collected data that served the purposes of the study. Second, we paraphrased the tweets we used as examples and removed all potentially identifiable information, such as names and locations, included in the tweets.

## 3. Results

As of the date on which the data were extracted, the median number of tweets per week per user was 21.0. Our analysis of tweets with #StopAsianHate revealed five themes: (1) Asian hate is not new; (2) Address the harm of racism; (3) Get involved in #StopAsianHate; (4) Appreciate the AAPI community’s culture, history, and contributions; and (5) Increase the visibility of the AAPI community. The themes, categories, codes, and example tweets are summarized and presented in Supplementary Table S1.

### 3.1. “Asian Hate Is Not New”

This theme highlights the importance of viewing the recent surge of COVID-19 related hate and discrimination against AAPIs from a historical perspective. With tweets such as “Anti-Asian hate isn’t new” and “[the AAPI community has] long been suffering from racial discrimination,” tweeters reviewed the legacies of anti-AAPI racism by reflecting on the history of racism against the AAPI community, sharing their personal or family’s lived experiences of discrimination, and spelling out all forms of racism and discrimination (e.g., “Violence, discrimination, and bigotry”). One tweeter shared, “My mom was told by her client to ‘go back to your country’ … This is the sad reality for many, many Asian-Americans.”

Many tweets acknowledge that the COVID-19 pandemic has provided an opportunity for established, underlying currents of anti-Asian hate and other forms of racism to surface, often violently. Tweeters shared alarming statistics, as described in the following tweets: “Hate crimes against AAPI New Yorkers increased by 833% from 2019 to 2020” and “the actual number can be much higher since a lot of the incidents were unreported.” Tweeters also expressed that vulnerable groups in the AAPI community were disproportionately affected, including women and older adults; for example, “Almost 4 in 5 AAPI women have been impacted by hate crime as they were faced with twice the risk to be targets of anti-Asian hate.”

### 3.2. “Address the Harm of Racism”

This theme presents the detrimental consequences of racism on the AAPI community and addresses efforts to curtail such consequences. Besides the physical harm caused by racism against AAPIs, the mental health burden of racism was a shared concern across tweets, as exemplified by the following tweet: “[physical harm by racism] is leading to a mental health crisis in our community.” Tweeters noted that in addition to other obstacles, the stigma associated with mental health issues hindered the AAPI community’s needs from being adequately recognized and addressed. As one tweeter explained, “Now is the time to learn about the mental health stigma as well as the structural and cultural barriers preventing Asian-Americans from seeking mental health care.”

Although the scope of the harm created by racism remains challenging, tweeters shared relevant efforts and recommendations regarding resources to address it. Some resources pertained to useful responses to Asian hate, such as self-defense and help centers and services (e.g., victim services center, chat service). Users also shared information about diverse programs resulting from community efforts to combat violence and ensure safety, including “Public Safety Patrol,” “Neighborhood Safety Companions,” “Group of volunteer Chinatown Safety Ambassadors,” “Volunteer program in Chinatown for older adults”, and “Yellow Whistle project”. Recommendations on how to improve AAPI mental health were shared, and the need for culturally competent care was emphasized. As one tweet stressed, “The need for culturally competent mental health care by and for AAPI communities is increasingly evident”.

### 3.3. “Get Involved in #StopAsianHate”

Tweeters called for a dedicated effort to combat Asian hate by joining the #StopAsianHate movement. They believed that Asian hate should be considered and treated as a shared responsibility across different racial/ethnic groups, and that discussion and efforts at individual and societal levels were needed to tackle racism and promote diversity and inclusion.

To strengthen awareness of the shared responsibility of addressing Asian hate, the tweeters asked their readers to work in solidarity by building allyship with other marginalized communities. Some tweets pointed out that “Only solidarity can anchor us at a time of increasing anti-Asian hate and persistent violence targeting other minority groups such as Black, indigenous and Latinx communities” and “We encourage everyone to become an ally. Advocacy and allyship is the only way we can fight against ignorance, intolerance, and hate”. As part of the solidarity efforts, tweeters also encouraged people to support the AAPI community with a variety of approaches, such as by expressing “support, camaraderie, and love for our communities” and supporting local AAPI businesses.

Tweeters discussed current efforts as well as future directions to address Asian hate at the individual and societal levels. At the individual level, soft power such as art (e.g., artwork, comedy, poetry, and performance art) by members and allies of the AAPI community was recognized as crucial to (a) raise public awareness toward discrimination and violence against AAPIs, (b) facilitate the #StopAsianHate movement, and (c) uplift the AAPI community. As one tweeter shared, “What a wonderful image with a powerful message! We are ALL human beings and deserve to be treated as humans regardless of race, gender, or sexual preference.” In addition, many tweeters leveraged entertainment media and pop culture to express support for AAPIs in ways that were authentic and had crossover appeal. Tweeters encouraged individuals to contribute to the #StopAsian Hate movement through (a) sharing personal stories and experiences of movement involvement, (b) engaging in fund-raising activities, (c) showing or wearing actionable items such as a gold ribbon, and (d) sharing support on social media. Besides commenting on immediately actionable items, many tweeters shared their thoughts on actions for long-term, sustainable impact. Their recommendations included reflecting on one’s own bias and prejudice by “Step[ping] back, look[ing] in a mirror, do[ing] the work to check yourselves and grow”; taking advantage of resources to prevent hate crimes and bias incidents (e.g., bystander intervention or training to stop xenophobic harassment); and showing support through daily actions (e.g., “speak up”, “keep pushing”, “continue to fight”).

As for societal-level efforts, tweeters were generally positive about the significance of the COVID-19 Hate Crimes Act as “a crucial step towards confronting the rising tide of anti-Asian attacks” while also recognizing that additional governmental support and legal protection is needed to “address the root causes of systemic racism and oppression”. Furthermore, users viewed education as a fundamental approach to confronting anti-AAPI racism; this could take the form of establishing AAPI history curriculum in schools, educating leaders in power on anti-AAPI racism, and increasing public awareness of different forms of racism such as the “model minority myth” and microaggressions challenging AAPIs.

In addition to directly addressing Asian hate, tweeters also expressed an overall need to promote diversity and inclusion across different fields, including sports, business, and the entertainment industry. Some expressed that one of the most fundamental ways to facilitate diversity and inclusion is through language use. Examples of such efforts included setting up support services for victims in multiple languages or sharing practical guidance. Others introduced tips for diversity in workplace culture, and many called for social reform and a more significant commitment to diversity, equity, and inclusion work within organizations.

### 3.4. “Appreciate the AAPI Community’s Culture, History, and Contributions”

Tweeters voiced the importance of appreciating diverse cultures, learning their rich histories, and acknowledging the APPI community’s contributions. They shared relevant educational resources highlighting the diverse cultures and ethnic heritages of the AAPI community. For example, one tweeter discussed the unique meaning of food in APPI culture: “Food is the language for love for our AAPI community. Food is what we use to cope with the pandemic and the surge of hate”. Tweeters also advocated learning about AAPI history in order to understand the historical roots of anti-AAPI racism and contextualize the recent rise of anti-Asian sentiment. They felt it is especially important for “the younger generation to learn more about the history and experience of Asian communities”. They emphasized the contributions made by the AAPI community to various industries, as AAPIs are “deeply woven into the fabric of this country [America].” Specifically, tweeters celebrated AAPI community members for their efforts as health care professionals who were confronted with racism while saving lives in health care settings during the COVID-19 pandemic. As an example, one tweeter shared that “We celebrate and stand with AAPI healthcare workers. We will fight for a safe environment where they are empowered, and they can thrive!” In addition, AAPI organizations who fought for racial equity were also celebrated.

### 3.5. “Increase the Visibility of the AAPI Community”

This theme details the invisibility of AAPIs across multiple life domains and emphasizes that “representation matters”. AAPI tweeters posted about the challenges of being unheard and often neglected in regard to “our needs, achievements or suffering”. Not only was this neglect evidenced in daily life, but by the lack of APPI data in federal and state statistics and research support, as exemplified by one tweet: “Asian Americans must be seen when it comes to health equity while NIH and HHSGov spends less than 1% of its funding on our communities”. Tweeters argued that AAPI representation within many different systems and on numerous platforms is needed (e.g., in education, politics, the media, and especially in leadership), and that without representation, the AAPI community would not “have a voice in advocating for equity”. Achieving data equity was recognized as essential because AAPI voices are “missing from the national dialogue”. The tweeters encouraged the AAPI community to contribute to research in order to “amplify (their) voices to the world” and advocated for the data collection on hate crimes to “fill the gap in AAPI data”.

## 4. Discussion

The current study used thematic analysis to examine the contents of Twitter as it relates to racism against AAPIs using the hashtag “StopAsianHate”. Importantly, our analysis of #StopAsianHate tweets has revealed five themes.

First, tweeters note although it is gaining increasing national attention, the community’s current plight is not new (“*Asian hate is not new*”) as acts of racial discrimination have been committed against the AAPI community throughout history. Although not detailed in our results, such acts include the Chinese Exclusion Act (1882), prohibiting the immigration of Chinese laborers into the United States; the Page Act (1875), America’s first immigration ban based on race; the destruction of Chinatown (1887); and the murder of Vincent Chin (1982). They also tweeted a series of documentaries on their family histories and personal experiences with racial bias rooted in anti-Asian racism. As noted by Quint [23], highlighting issues related to legacies of hate/racism against AAPIs can help them to center their unique needs and experiences and address the multiple and intersecting barriers and hardships they face. Tweeters also acknowledge that underlying racism has resurfaced during the pandemic. Epidemic-provoked hate and discrimination is a critical issue. According to one article [24], between March 2020 and March 2021, the “Stop AAPI Hate” reporting center received over 6600 reports of hate incidents directed at AAPIs such as verbal harassment, shunning, and physical assault. The New York City Police Department also reported a 1900% increase in anti-Asian hate crimes in the year 2020 alone [25]. In particular, AAPI women and girls were reported to be the primary targets, which our data also show.

Given the current surge of anti-Asian hate, tweeters raised awareness about the physical and mental harm caused by racism (“*Address the harm of racism*”). Indeed, research has shown that AAPIs who have experienced COVID-19-related discrimination report higher levels of anxiety and depression, and sleep problems [26]. The surge of racism could also have long-term health effects [27] as chronic stress induced by frequent racist encounters can increase the risk of post-traumatic stress disorder, heart disease, high blood pressure, diabetes, inflammation, and other illnesses. However, some tweeters acknowledge that finding and obtaining help is not always a straightforward process due to structural and cultural barriers. Growing evidence shows that AAPIs are less likely to access mental health services than any other racial group due in part to a cultural stigma surrounding mental health and a lack of culturally relevant approaches to treatment [28,29]. Existing research on this issue suggests strengthening training for providers to help them deliver culturally competent care [30,31] as well as providing interventions that may carry less cultural stigma than one-on-one therapy, such as psychoeducational workshops or support groups. At the same time, psychologists are encouraged when designing interventions to look beyond traditional therapy and to study the racist incidents themselves, including their root causes and outcomes, in order to identify systemic issues and not just individual pathology [32].

In addition, tweeters encourage action against Asian hate as part of the #StopAsianHate movement (“*Get involved in #StopAsianHate*”) in several ways, among which “intersectional solidarity” stands out. Cheng et al. [31] argue that intersectional solidarity synergistically confers collective psychosocial resilience against COVID-19 anti-Asian racism. Although not detailed in our results, the present study showed that the Blasian March (i.e., a solidarity action between Black/African, Asian, and mixed Blasian communities) received much attention, and this confirms the observations of Lyu et al. [33]. Just as importantly, some users argued that overcoming hate requires efforts beyond marching or protesting, such as changing feelings and attitudes through art. Besides these individual efforts, tweets highlighted many upstream efforts. For example, tweeters emphasized the importance of confronting racism through education. Although not detailed in our results, they advocate for the inclusion of AA history in K–12 curriculums, highlighting exemplary work such as the Teaching Equitable AA Community History (TEAACH) Act. The TEAACH Act requires public schools in Illinois to teach a unit of AA history that covers the history of AAs in Illinois and the Midwest and the role played by AAs in advancing civil rights [34,35]. This movement is critical [35]. Despite their important role in building America, the many contributions of AAs are just starting to be taught in schools [36]. Although research suggests that teaching children about their racial and ethnic identity helps them to recognize societal discrimination, there is hesitancy to make school curriculums more inclusive [37].

The #StopAsianHate movement also led to a surge in tweets encouraging the younger generation to learn more about AAPI cultures and histories (“*Appreciate AAPI community’s culture, history, and contributions*”). Particularly, it is noteworthy that there has been a heavy presence on Twitter of educational resources that address AAPI issues and topics, uniquely enriching identities and cultures, and community histories in order to further understand the current rise in anti-Asian racist incidents and attitudes. Tweeters also emphasized the importance of recognizing the contributions of AAPIs to America. Notably, many users posted that the remarkable acts of support and solidarity among health care and essential workers, including nurses, were the driving force behind the maintenance of the health care industry during COVID-19.

The last theme of the #StopAsianHate movement relates to calling for greater visibility of Asians across sectors (“*Increase the visibility of the AAPI community*”). Despite being the fastest-growing racial/ethnic group in the U.S., 23 million AAs lack visibility in American politics, corporations, media/culture, arts, sports, and other communities [38,39]. Although not detailed in our results, tweeters particularly stressed a need to support AA leaders, promote Asian participation in authentic hiring for positions of power, increase AA voter turnout, and showcase AA influencers in order to embrace Asian voices. Furthermore, it is important to note that many users looked to data or evidence as an important part of advocacy and encouraged the public to complete surveys in order to fill the gap in AAPI data and amplify the voices of AAPIs. As can be seen in our example tweets, many users tweeted the Young Asian American Health Survey (https://www.yaahsteam.org), a national survey on the mental health and well-being of young AAs amid the rising anti-Asian racism during COVID-19, arguing that no one should be under the illusion that anti-Asian hate only affects older immigrants. Wu [40] notes that although a growing number of studies address gender and racial/ethnic biases, there is a lack of data on AAPI youth, and young adults are highly vulnerable to the negative effects of racism on mental health and identity development. Additionally, tweeters highlight the importance of collecting data on hate crimes; relevant data has long been underreported because distrust of law enforcement, language barriers, and immigration status are deterrents to reporting a crime [41]. In addition, law enforcement agencies often aggregate reports by ethnicity, lumping incidents against specific groups into broader racial categories [42], which can obscure important patterns and unique challenges encountered by racially minoritized groups such as AAs.

### Limitations & Future Directions

Our study has several limitations. First, we analyzed English-language discussions only, which could have led to certain biases. Second, due to the nature of the tweet data, we were unable to provide the profiles of the users, but we acknowledge that tweeters’ demographics might influence the content of their tweets. For instance, one survey revealed that nearly half of tweeters are young, well-educated, mobile adults, whose views do not necessarily reflect those of the broader public [43]. Third, we may have missed relevant information due to the inaccessible tweets at the time of data extraction. Fourth, Twitter data represent only what people are comfortable expressing online and in the public sphere; therefore, collected discussions may differ from in-person discussions and interactions. Fifth, although we believe that the tweets used in this study provided sufficient information for our analysis, we did have to remove many tweets that were not relevant to the hashtag #StopAsianHate during screening. This reduced the total data available for analysis, while these tweets would not have further enriched our results. Twitter is often riddled with computer-generated spam, as well as messages containing irrelevant information, misspelled words, or unorthodox abbreviations. This is considered one of the major challenges in data extraction from Twitter [43]. Sixth, the relatively short time period covered by our analysis is another limitation. Our data were gathered from the 14-day period right after the COVID-19 Hate Crimes Act was signed into law. We chose this time frame in order to best discern people’s responses to this event, when tensions around these issues were heightened. However, social media is very dynamic, and the tone of discussions and the common understanding of events can change rapidly. Subsequent events can shift the focus of tweets in new directions. Consequently, the next essential step in this work will be to capture the long-term trends in online discourse related to the #StopAsianHate movement. In addition, future validation studies should explore whether sentiments expressed in our data translated to action and how long they persisted. Finally, a more complete study is warranted considering other social media.

## 5. Conclusions

By taking a data-mining approach, we examined the contents of Twitter as it relates to racism against AAPIs using the hashtag “StopAsianHate.” Several lessons can be learned from the present findings, which can serve as a foundation for future evidence-based strategies for addressing anti-AAPI racism and prejudice. First, there is a generational shift between older AAs who might have once said: “swallow the bitterness” of disrespect and hate and those who now say #Rise up and #SeeUsUnite to #StopAsianHate. We gather that Twitter is a useful and influential platform for spreading awareness, which plays a crucial role in the public’s perceptions and significantly influences the public’s communication during a crisis. Second, we learned there is an urgent need for culturally competent care delivery that embraces culturally relevant interventions, psychosocial support services, and mental health risk assessments for AAPIs. Service providers should be aware of the broader social context in which racism is rooted and the systemic biases and inequities driving psychological distress in AAPI individuals to provide more culturally sensitive psychological care. Third, tweet data provided a window into issues of education. Tweeters recognized that education is one part of a “multipronged” strategy to tackle the rise in discrimination against AAPIs. They were raising awareness, building understanding, and broadening educational efforts about AAs. Further, Twitter was used as a public education tool to tackle harmful stereotypes underlying public opinion about AAs, such as the “model minority myth.” Fourth, apart from “hard power” (e.g., federal hate crimes legislation, push for tougher laws) as a combat strategy, it is interesting to note that the use of “soft power” (e.g., art, knowledge) to inspire and educate the world about AA tradition, history, culture, and celebration as well as raise awareness of discrimination and violence stands out. Last, throughout the #StopAsianHate movement, an important piece of advocacy for AAPIs has been data. People place heavy significance on AAPI data, arguing that, without data, AAPIs remain invisible to policymakers, philanthropists, and decision-makers.

## Data Availability

The data presented in this study are available on request from the corresponding author.

## Supplementary Materials

The following supporting information can be downloaded at: https://www.mdpi.com/article/10.3390/ijerph19073757/s1, Table S1. Summary of themes, categories, codes, and example tweets shared in #StopAsianHate.
