# Supplementary material for: The #StopAsianHate Movement on Twitter: A Qualitative Descriptive Study
Source: Int J Environ Res Public Health. 2022 Mar 22;19(7):3757. doi: 10.3390/ijerph19073757 (PMC8997488; doi:10.3390/ijerph19073757)
Supplement: Supplementary file 1 [file ijerph-19-03757-s001.zip › ijerph-1580904-supplementary.pdf]

International Journal of
Environmental Research and Public Health

MDPI

Supplementary Table S1. Summary of themes, categories, codes, and example tweets shared in #StopAsianHate.

Themes	Categories	Codes	Example tweets
**Asian hate is not new**	**Legacies of hate/racism against AAPIs**	Histories of racism, struggles, prejudice, or violence in AAPI communities	"Asian Americans have been suffering from racial discrimination for a long time as a result of both the habitual inaction of the government and a racist social system."
		Personal (or family) stories of discrimination or challenges	"My mom was told by her client to 'go back to your country'… This is the sad reality for many, many Asian-Americans."
		All forms of discrimination including stigmatization, racism, prejudice, implicit bias, or institutional racism	"Violence, discrimination, and bigotry are persistently affecting all minorities… we painfully witnessed Asian Americans were targeted during the pandemic. The racial discrimination comes in all shapes and is everywhere. We should remember that fighting anti-Asian hate can never be separable from fighting racism in all its forms."
	**Epidemic-related hate/discrimination**	Increased anti-Asian racism toward vulnerable groups such as women or older adults	"Most incidents happened in public areas (e.g., businesses or streets). One tenth of the incidents happened online. Almost 4 in 5 AAPI women have been impacted by hate crime as they were faced with twice the risk to be targets of anti-Asian hate."
		Raise awareness of hate crime or violence against AAPIs	"From March 2020 to March 2021, 6,603 hate incidents were documented by StopAAPIHate while the actual number can be much higher since a lot of the incidents were unreported."
**Address the harm of racism**	**Harm of racism**	Physical harm caused by racism	"For Asian Americans, the physical violence by racism is leading to a mental health crisis in our community."
		Mental health burden of AAPIs	"1 out of 5 Asian American victims of racism experienced racial trauma as well as elevated level of mental health symptoms, such as depression, anxiety, stress, and physical symptoms."
	**Efforts to address harm of racism**	Sharing resources on responding to Asian hate	"XX Organization is now offering workshops on self-defense for the elderly as they were severely attacked or even killed. You will learn practical skills to react to physical attacks: http://XXX."
		Community efforts to combat violence and ensure safety	"A volunteer patrol group in Chinatown has motivated Asian Americans to be the strongest advocate for themselves to combat Asian hate after the recent rise in violence against American community. http://XXX"

		Improve mental health for AAPIs	"We are proudly standing with the AAPI community to fight hate crimes! Please call us at XXX and our psychologists will provide mental health support to you whenever you need."
**Get involved in #StopAsianHate**	**Shared responsibility**	Fighting in solidarity	"We encourage everyone to become an ally. Advocacy and allyship is the only way we can fight against ignorance, intolerance, and hate."
		Support local AAPI business	"It is never easier to support AAPI business, thanks to the initiative by X organization. AAPI business were disproportionately affected by COVID-19. I can't wait to support AAPI businesses."
		Show support to AAPI communities	" 'Stop Asian Hate' is a cry that needs to be heard by the whole world. No one should ever go through what Asian people are going/have been going through.' - Oscar Mar"
	**Individual-level efforts**	Use soft power such as art	"What a wonderful image with a powerful message! We are ALL human beings and deserve to be treated as humans regardless of race, gender, or sexual preference."
		Encourage others to get involved in anti-Asian racism movement	"Use social media as a way to let your voice heard. Let's raise awareness about anti-Asian racism. Use #XXX to keep our conversation going."
		Recommendations for sustainable action	"We need [to] continue our support for the AAPI community and fight for what's right. Change and activism are not a trend. Let's keep educating, donating, and supporting local AAPI businesses."
	**Societal-level efforts**	COVID-19 Hate Crimes Act	"Hate crimes continue to target [the] most vulnerable of our Asian Americans in spite of the passage of the COVID-19 Hate Crimes Act. We must continue to fight against bigotry against our communit [as] communities in solidarity."
		Call for governmental support and legal protection for AAPIs	"Violence against Asian[s] goes beyond [the] individual level and is rooted in how our country is governed. For example, we have to hold the government accountable when it comes to how it enacts anti-Asian violence through deportation."
		Stress the importance of standing up to racism through education	"All of us [are] responsible for contributing to an inclusive environment that celebrat[es] the AAPI community. We can do that from education to gain a cross-cultural understanding."

	**Promoting diversity & inclusion**	Use of appropriate languages to show support	"We need to have a better understanding of [the] diverse AAPI community in honor of the Heritage Month. Use the proper language to show your support and become a better ally. Learn more here: https://XXX."
		Promote cultural diversity & inclusion in sports, business, and entertainment	"We appreciate the diverse backgrounds, lived experiences and cultures within the communities we serve. We are advocators for equal opportunities for all."
		Tips to support workplace diversity	"Racism often exist[s] as microaggressions in workplaces. When you witness a microaggression, NEVER do the following: Don't act like you didn't hear or see it. Don't make excuses. Don't become immobilized. Instead, DO the following: Address the microaggression by responding with a non-judgmental observation or asking a thoughtful question. Talk to those involved."
**Appreciate the AAPI community's culture, history, and contributions**	**Importance of learning about AAPI culture**	Share educational resources to learn about AAPI heritage and culture	"Food is the language for love for our AAPI community. Food is what we use to cope with the pandemic and the surge of hate. Members from the AAPI community including chefs and celebrities shared the role of food in their community to celebrate the AAPI Heritage Month: http://XXX."
		Promote the understanding of unique cultural and ethnic heritage	"Check out the educational resources to learn more about AAPI heritage and culture: http://XXX"
	**Importance of history of AAPI communities**	Encourage the younger generation to learn about the history and experiences of AAPI communities	" 'We need to make sure that all Americans learn Asian American history and the history of all ethnicities. We need institutional changes in our country to StopAsianHate crimes.'- Congressmember Judy Chu"
		Understand the historical roots to contextualize current racism/hate against AAPI	"We will never understand the anti-Asian racism and hate in the current moment unless we understand the history of exclusion."
	**Acknowledge the contributions of AAPIs**	Efforts made by AAPI community members	"Our contribution is deeply woven into the fabric of this country. The contributions and pleas for safety of APPI community won't be ignored."
		Support AAPI healthcare workers who fight COVID and confront racism/racial bias in healthcare settings	"We celebrate and stand with AAPI healthcare workers. We will fight for a safe environment where they are empowered and they can thrive!"
		Support AAPI organizations that fight for racial equity	"We are committed to stop AsianHate by supporting the following organizations: XX, XX, and XXX."

**Increase the visibility of the AAPI community**	**Invisible Asians**	Asian voices are usually going unheard and neglected	"Our community are invisible, especially our needs, achievements or suffering, to American society. We become visible when they want to assault someone or use a stereotype."
		Lack of APPI data in federal/state statistics/research support/funding	"Asian Americans must be seen when it comes to health equity while NIH and HHSGov spends less than 1% of its funding on our communities: http://XXX. "
	**Visibility/representation matters**	Promote Asian representation and participation in the education system, media, and politics	"AAPI community members are usually overlooked in leadership positions and are faced with multiple barriers to promotion and career development. AAPI will not have a voice in advocating for equity if they are not able to sit at the leadership table."
		Data equity	"The mainstream film industry continues to lack APPI representation according to a new study. Only 5.9% of the 51,159 speaking characters were APPI, less than the 7.1% of the U.S. population who self-identified as AAPI."
